# ORAL HEALTH AND COGNITION AMONG U.S. COMMUNITY-DWELLING CHINESE OLDER ADULTS

**DOI:** 10.1093/geroni/igz038.1269

**Published:** 2019-11-08

**Authors:** Darina V Petrovsky, Bei Wu, Weiyu Mao, XinQi Dong

**Affiliations:** 1 University of Pennsylvania, Philadelphia, Pennsylvania, United States; 2 New York University Rory Meyers College of Nursing, New York, New York, United States; 3 University of Nevada, Reno, Reno, Nevada, United States; 4 Rutgers University Institute for Health, Health Care Policy and Aging Research, New Brunswick, New Jersey, United States

## Abstract

The purpose of this study was to examine the associations between tooth/gums symptoms and changes in cognitive function. We used data from the Population Study of Chinese Elderly in Chicago, a two-wave epidemiological study of 2,713 U.S. Chinese older adults. We selected self-reported oral (tooth and gum) symptoms as independent variables. We measured global function and three cognitive domains: episodic memory, executive function and working memory. Adjusting for sociodemographic and health-related characteristics, participants who reported having teeth symptoms at baseline, experienced their global cognition and episodic memory decrease (both p<0.05). Participants who reported having teeth symptoms at baseline, experienced a faster rate of decline in global cognition for every additional year. However, this effect disappeared once we adjusted for all covariates. We found no significant relationship between baseline gum symptoms and change of cognitive function. Future research directions, clinical and policy implications will be discussed.

